# Tropical Cellulolytic Bacteria: Potential Utilization of Sugarcane Bagasse as Low-Cost Carbon Source in Aquaculture

**DOI:** 10.3389/fmicb.2021.745853

**Published:** 2021-10-29

**Authors:** Wei Ren, Xueni Xu, Hao Long, Xiang Zhang, Xiaoni Cai, Aiyou Huang, Zhenyu Xie

**Affiliations:** ^1^State Key Laboratory of Marine Resource Utilization in the South China Sea, Hainan University, Haikou, China; ^2^Hainan Provincial Key Laboratory for Tropical Hydrobiology and Biotechnology, Hainan University, Haikou, China; ^3^College of Marine Sciences, Hainan University, Haikou, China; ^4^Laboratory of Development and Utilization of Marine Microbial Resource, Hainan University, Haikou, China

**Keywords:** tropical cellulolytic bacteria, sugarcane bagasse, low-cost carbon source, hydrolysis capacity, multiple antibiotic resistance, decomposition, recirculating aquaculture system

## Abstract

Sugarcane bagasse (SB), as a major by-product of sugarcane, is one of the most abundant organic matter and characterized by cheap and easily available carbon source in Hainan Island, China. The objective of this study was to isolate tropical cellulolytic bacteria from Hainan Island and demonstrate their prospects of utilization of SB as a low-cost carbon source to greatly reduce the cost of aquaculture. A total of 97 cellulolytic marine bacteria were isolated, of which, 58 cellulolytic marine bacteria displayed the hydrolysis capacity (HC) of more than 1, while 28 cellulolytic marine bacteria displayed more than 2. Of the 28 tropical cellulolytic bacterial strains with HC more than 2, *Microbulbifer* sp. CFW-C18 and *Vibrio* sp. MW-M19 exhibited excellent SB decomposition in a small-scale laboratory simulation of shrimp aquaculture, up to 75.31 and 74.35%, respectively, and both of them were safe for shrimps. Meanwhile, both of CFW-C18 and MW-M19 besides displaying low multiple antibiotic resistance (MAR) index, also increased the C/N ratio (CFW-C18: C/N ratio of 14.34; MW-M19: C/N ratio of 14.75) of the small-scale laboratory simulation of shrimp aquaculture by decreasing the nitrogen content after a supplement of SB for 15 days. More importantly, CFW-C18 and MW-M19 displayed a relatively low MAR index, 0.47 and 0.1, respectively, especially MW-M19, with the lowest MAR index (0.1), which was resistant to only three antibiotics, streptomycin, amikacin, and levofloxacin, indicating that this strain was safe and non-drug resistance for further use. Overall, tropical cellulolytic bacteria isolated from Hainan Island, especially CFW-C18 and MW-M19, will provide the proficient candidates as probiotics for further construction of the recirculating aquaculture system based on the supplement of low-cost external carbon source—SB.

## Graphical Abstract

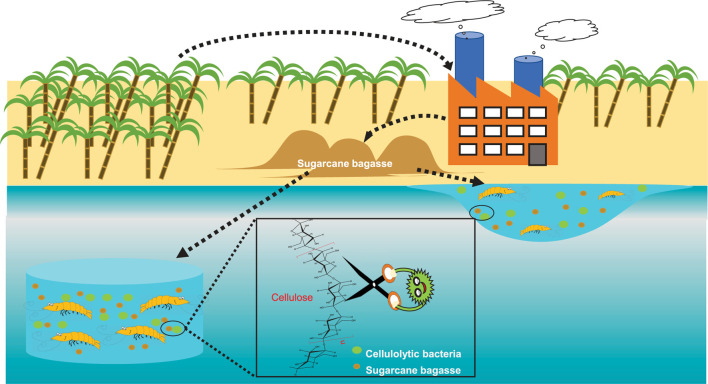

## Introduction

*Litopenaeus vannamei* aquaculture, as an important food production, has become one of the pillar industries in the agricultural economy of most coastal countries, which is considered a good alternative because of its high economic value, delicious meat, rich nutrition, low feed conversion rate, high survival rate, low water consumption, short growth cycle, etc., (Hoang et al., 2020; Huang et al., 2020). With the rapid development of aquaculture technology, the mode of *L. vannamei* aquaculture experienced a gradual change from extensive aquaculture to intensive aquaculture. However, intensive aquaculture has been restricted by water eutrophication, such as nitrogen/phosphorus enrichment and organic deposition, which indirectly led to harmful algal blooms (Kuhn et al., 2010; Guo et al., 2016; Li et al., 2020; Wei et al., 2021a), of which the accumulation of ammonia–nitrogen and nitrite–nitrogen is of immense importance for water quality in intensive aquaculture. Ammonia–nitrogen is mainly derived from protein-rich bait, which will be conversed to nitrite–nitrogen by the nitrifying action of aquatic microorganisms (Ren et al., 2021). To pursue high-yield/income, some operations blindly increased the scale of aquaculture and put in bait and fertilizer without efficient utilization by the aquaculture system, then the accumulated biological excreta and inedible feeds in the aquaculture system were decomposed into high concentrations of nitrogen (ammonia–nitrogen and nitrite–nitrogen), phosphorus, and organic waste resulting in the decrease in C/N ratio, thereby making it difficult for aerobic microorganisms to survive since the decomposition of organic matter consumed a great deal of oxygen, and inhibiting the assimilation of heterotrophic microorganisms (Lin and Chen, 2001, 2003; Hoang et al., 2020; Mariane de Morais et al., 2020; Penalosa-Martinell et al., 2020). Actually, nitrogen that can be used by aquatic animals is very limited in the processing of aquaculture, and the nitrogen of the protein-rich bait deposited in the bottom mud will make the water turn black. We reason that the content of ammonia–nitrogen and nitrite–nitrogen will decrease with an increase in C/N ratio, whereas bacterial de-nitrogen rate will increase with that.

Currently, biological treatments in the tail water of aquaculture have become a research hotspot. Of which the supplement of a carbon source (mainly including sucrose and molasses) is a useful method to reduce nitrogen in an aquaculture system, which is a practical method to control inorganic nitrogen, promote the growth of heterotrophic bacteria, enrich the diversity of bacteria, improve the aquaculture environment, and increase the total production of aquaculture by controlling C/N ratio in the aquaculture system (Azim and Little, 2008; De Schryver et al., 2008; Dawood et al., 2018; Hisano et al., 2019). If the C/N ratio in water is unduly high, a part of the nitrogen will be used by bacteria to synthesize protein. Reportedly, bio-floc technology provides a mode to construct marine recirculating aquaculture system by the supplement of a carbon source, which can promote the growth of heterotrophic bacteria, remove inorganic nitrogen from the water, and maintain water balance (Azim and Little, 2008; Hisano et al., 2019; Poli et al., 2019). The process mainly transforms nitrogen into protein required by microorganisms as the source of feed protein for aquatic animals to avoid the accumulation of inorganic nitrogen in the water body. The exogenous carbon sources can promote the growth of microorganisms in aquaculture and provide the required carbon source to form bio-flocs, thereby meeting the ingestion of aquatic animals, and reducing the accumulation of toxic inorganic nitrogen compounds (Avnimelech, 1999; Burford et al., 2004; De Schryver et al., 2008). Therefore, it is an effective method to improve the yield of *L. vannamei* and solve the water pollution by adding exogenous carbon sources (mainly including sucrose, glycerin, glucose, and sodium acetate) in the aquaculture system to adjust the C/N ratio (Grommen et al., 2002; Samocha et al., 2007). However, these carbon sources are expensive and can easily cause environmental pollution.

Sugarcane is a widely grown sugar crop in Hainan, China, and sugarcane bagasse (SB) as a major by-product is the main waste in the sugar industry. Generally, about 260–280 kg of wet SB can be produced per ton of sugarcane, with a total production of more than 279 MMT tons annually worldwide (Dong et al., 2017; Xu et al., 2019). About 40–50% of dried SB is cellulose, whereas it is very difficult for this polysaccharide to decompose because of its stable chemical linkages inside a monomeric cellulose chain, as well as the crystalline structure formed by multiple cellulose microfibrils that are interconnected by hydrogen bonds (Liu et al., 2019). Cellulose is the most abundant organic matter on the earth and characterized as a cheap and easily available carbon source. Microbial cellulolytic enzymes, called cellulase, are complex enzymes that consist of endoglucanases, exoglucanases, and β-glucosidases, which synergistically work to hydrolyze the β-1,4 glycosidic bonds of cellulose (Wang et al., 2020). Most cellulolytic enzymes with high activity used in commercial applications are produced from fungi (Hu et al., 2017), whereas bacteria have higher growth rate and enzyme production rate than that of fungi (Ren et al., 2016). More importantly, bacteria have higher thermal stability and genetic stability than that of fungi (Harms et al., 2011). Additionally, oceans covering more than three quarters of the surface of the Earth, is an open ecosystem, whereas at least 50%, and potentially more than 90%, of all marine species are undescribed (Ren et al., 2018, 2019a, b). Marine enzymes secreted by marine microorganisms are characterized by salt tolerance, hyperthermostability, barophilic, alkali resistance, and low optimum temperatures, which are necessary for efficient bio-process exploitation (Ren et al., 2018, 2019a, b). Hainan is a big maritime province in China and rich in marine resources, but the knowledge of cellulolytic microbes isolated from this area is extremely limited. Accordingly, it is urgent to find tropical cellulolytic bacteria for long-term utilization of SB as a low-cost carbon source, which will greatly reduce the cost of aquaculture in Hainan, even around the world. Therefore, the purpose of this work was to determine competent cellulolytic marine bacteria for SB decomposition as the low-cost external carbon source in an *L. vannamei* mariculture system.

## Materials and Methods

### Description of Sampling Sites and Sample Collection

The shrimp and water samples were collected from 0.5-m deep, around 1 m away from the aerator of an aquaculture pond, and the muddy sediment samples were extracted at a sediment depth of 3-cm deep along the seagrass. All samples were from four sites located in Wenchang, Hainan Island, China: two shrimp culture bases in Haiwei town (19°26′39.02″N, 108°50′11.23″E) and Huiwen town (19°27′28″N, 110°45′13″E), seagrass beds around the mangrove in Huiwen town (19°28′11.66″N, 110°47′41.22″E), and seagrass beds in Dongjiao Coconut Forest, Huiwen town (19°31′28.81″N, 110°52′0.45″E). The shrimp and mud samples were placed in sterile zipper plastic bags, and the water samples were stored in sterile sampling bottles. All of the samples were stored at 4°C for the isolation of marine bacteria (Supplementary Table 1; the details of the methods and results are presented in the Supplementary Material).

### Screening and Identification of Cellulolytic Bacteria

Screening of the cellulolytic bacteria from marine bacteria was conducted by carboxymethylcellulose (CMC) agar plate and Congo red staining method. Two microliters of overnight growth culture in the marine 2216E of each marine bacterial isolate was spot plated on CMC agar [0.2% NaCl, 0.5% CMC sodium salt, 0.67% Na_2_HPO_4_, 0.13% (NH_4_)_2_SO_4_, 0.05% MgSO_4_⋅7H_2_O, and 1.7% agar]. The agar plates were incubated at 30°C for 96 h and then flooded with 1 mg/ml of Congo red for 30 min at room temperature. The cellulolytic isolates were detected by the cellulolytic zone around the colonies after Congo red staining. The hydrolysis capacity (HC) value, which determined the cellulolytic activity, was calculated from the ratio between the diameter of the cellulolytic zone and the diameter of the bacterial colony. In this study, we only selected colonies with a ratio of greater than 1. The selected cellulolytic isolate was identified by molecular genetic analysis. The PCR amplification and 16S rDNA sequence analysis were performed using universal primers 27F (5′-GAGTTTGATCATGGCTCAG-3′) and 1492R (5′-CGGTTACCTTGTTACGACTT-3′) (Wei et al., 2021a). Bacterial sequences were compared with 16S rDNA reference gene sequences by BLAST^1^).

### Cellulolytic Activity Assay

The selected cellulolytic isolate was grown in CMC broth (0.2% NaCl, 0.5% CMC sodium salt, 0.5% tryptone, and 0.1% yeast extract) at 30°C for 24 h. Bacterial cells were removed from the culture broth by centrifugation at 5,000 rpm for 15 min at 4°C. The cell-free supernatant obtained after centrifugation served as a crude enzyme solution, which was analyzed for cellulolytic activity assay. Filter paper activity (FPase) was used to estimate the cellulolytic activity, and concentrations of the produced reducing sugars were estimated by the dinitrosalicylic acid method using glucose as the standard (Ren et al., 2018, 2019a, b). Briefly, cellulolytic activity was measured by incubating 0.5 ml of enzyme solution with 1 ml of substrate solution (a 1 × 6-cm strip of Whatman No.1 filter paper immersed in 1.0 ml of 100 mM sodium acetate buffer, pH 4.8) at 50°C for 30 min. The liberated reducing sugars were determined by the 3,5-dinitrosalicylicacid (DNS) method. The enzyme reaction was terminated by adding 3.0 ml of DNS reagent and then boiled for 5 min. The solution was completely cooled, and the optical density of the reaction mixture was measured at 540 nm. In order to evaluate the hydrolytic activity of cellulase on SB (SBase), only the filter paper was replaced by SB powder with a diameter of 0.05–0.1 mm as the substrate, and the other operations were the same. One unit of FPase/SBase was the quantity of enzymes that produced 1 μmol of glucose or reducing sugar per minute under the specified conditions. Additionally, based on the linear relationship between HC value and content of commercial cellulase from *Aspergillus niger* [24,000 U/g; TCI-Tixi Ai (Shanghai) Chemical Industry Development Co., Ltd., Shanghai, China], the cellulase production of bacterial strains were quantified (Supplementary Figure 1). All experiments of cellulolytic activity assay were performed in three replications, and the results were reported as mean ± SD.

### Small-Scale Laboratory Simulation Experiments

Before the experiments, juvenile shrimps, *L. vannamei*, were acclimatized for 2 months with continuous water exchange and constant aeration. The temperature and salinity were maintained at 28°C and 25‰, respectively. The shrimps were fed with commercial pellets (Yantai Dale Feed Co., Ltd., Yantai, China). Aeration was stopped for about 45 min during feeding, then feces and feed residues at the bottom were removed. Only 20% of the rearing water in each tank was replaced daily, and unhealthy or injured shrimps were removed. After the acclimation period, shrimps were randomly divided into two parts, one was for evaluation of the safety of cellulolytic bacteria in shrimp aquaculture, and the other was for evaluation of SB decomposition of cellulolytic bacteria in shrimp aquaculture. The shrimps in each treatment group were not fed for 1 day, and unhealthy or injured shrimps were removed. Finally, ensuring that the number of shrimps in each group was 10 by constantly replenishing healthy shrimp, the initial C/N ratio for each group was adjusted to about 10 by glucose. During the experiments, the temperature and salinity remained constant without replacing water.

In this study, we only selected colonies with an HC value greater than 2 (28 bacterial strains) to evaluate the safety of cellulolytic bacteria and SB decomposition of cellulolytic bacteria in shrimp aquaculture. The evaluation of the safety of the cellulolytic marine bacterial strains for shrimp was tested by immersion challenge with cellulolytic bacteria. The safety experiment for each strain was divided into two experimental groups, each having three replicates. The experimental group of 10 shrimps was immersed in the bacterial suspension at a concentration of about 10^7^ CFU ml^–1^, while the control group was immersed in rearing water without bacteria. All of the shrimps were fed basal diet and the mortality was monitored daily for 1 week. Meanwhile, the SB decompositions of cellulolytic bacteria was also evaluated in shrimp aquaculture, and the experiment for each strain was divided into three experimental groups, each having three replicates. The experimental group was immersed in the bacterial suspension at a concentration of about 10^5^ CFU ml^–1^ of rearing water containing 4 g/L of SB powder with an initial C/N ratio of approximately 12, while the control groups were immersed with rearing water containing 4 g/L of SB powder without bacteria with an initial C/N ratio of approximately 12 as positive control, and without the supplement of SB powder and bacteria with an initial C/N ratio of approximately 10 as negative control. All of the shrimps were fed basal diet, and the mortality, SB decomposition, and C/N ratio were monitored after 1 week. The rearing water was filtered by sterilized neutral detergent containing 37.2 g/L of EDTA, 13.6 g/L of osodium perborate, 30 g/L of SDS, 10 ml/L of glycol ether, and 23 g/L of Na_2_HPO_4_, and then washed by ethanol. The remaining SB (dry weight) in rearing water was used to measure the SB decomposition rate. Total carbon and total nitrogen were determined by Elemental Analyzer (Vario EL III produced by Elementar Analysensysteme GmbH, Langenselbold, Germany) (Wei et al., 2021b), which were used for the calculation of C/N ratio. Additionally, the SB decomposition of cellulolytic marine bacterial strains was also evaluated *in vitro*, and the details are presented in the Supplementary Material.

### Antibiotic Susceptibility Test

Antibiotic susceptibility tests were conducted by disk diffusion method, which were performed by spreading 0.1 ml of 18-h-old broth culture of the test strains on 2216E agar plates, and then placing antibiotics susceptibility disks (Wenzhou Kangtai Biotechnology Co., Ltd., Wenzhou, China) on the plates. Growth-inhibition zones around the disks were measured after incubation at 30°C for 19 h. In this experiment, 30 kinds of antibiotics belonging to 10 classes of antibiotic susceptibility disks were selected for antibiotic sensitivity tests with three replications (Supplementary Table 2).

Multiple antibiotic resistance (MAR) index of the present isolates against the tested antibiotics was calculated according to the following formula (Najiah et al., 2009):


MARIndex=X/(Y×Z)

where *X* = the total cases of antibiotic resistance, *Y* = the total number of antibiotics used in the study, and *Z* = the total number of isolates. A MAR index value of equal or less than 0.2 was defined as antibiotics that were seldom or never used.

## Results

### Tropical Cellulolytic Bacterial Profiles

Among the marine bacteria isolated from four sites in Hainan, 97 bacterial isolates were identified as cellulolytic bacteria since they exhibited a cellulolytic zone around their colonies on 2216E agar after Congo red staining (Supplementary Tables 1, 3 and Supplementary Figure 2; the details are presented in the Supplementary Material). Of the 97 marine bacterial strains, only 58 strains displayed HC values of more than 1, and all of them could secrete cellulase (Table 1). MW-C57 belonging to *Bacillus* had the highest FPase activity of 2.325 U/ml, while the FPase activities of most marine bacteria from this study areas ranged from 0.018 to 0.597 U/ml. Noteworthy, MW-M5 displayed the largest HC value (3.951), followed by MW-M10 (HC value = 3.752), and MW-M14 (HC value = 3.505). Additionally, there were six strains with HC values greater than 3, seven strains with HC values that ranged from 2.5 to 3, 15 strains with HC values that ranged from 2 to 2.5, and 29 strains with HC values that ranged from 1 to 2.

**TABLE 1:** FPase, relative enzyme content (REC), and hydrolysis capacity (HC) values of 58 cellulolytic bacteria.

Isolates code and species			Site	FPase (U/ml)	REC (U/g)	HC value
1	MW-C2	*Bacillus aquimaris*	D	0.017 ± 0.078	2.680 ±0.032	1.090 ± 0.038
2	MW-C3	*Microbulbifer* sp.	D	0.090 ± 0.104	3.107 ± 0.030	1.919 ± 0.171
3	MW-C4	*Microbulbifer* sp.	D	0.196 ± 0.244	3.077 ± 0.017	1.762 ± 0.056
4	CFW-C5	*Bacillus* sp.	D	0.017 ± 0.078	3.046 ± 0.010	1.672 ± 0.024
5	CFW-C6	*Bacillus safensis* sp.	D	0.288 ± 0.097	3.138 ± 0.004	2.187 ± 0.090
6	CFW-C7	*Bacillus subtilis*	D	0.342 ± 0.056	3.138 ± 0.002	2.138 ± 0.098
7	CFW-C8	*Bacillus* sp.	D	0.017 ± 0.078	3.117 ± 0.005	1.913 ± 0.024
8	CFW-C9	*Bacillus sp.*	D	0.145 ± 0.003	3.137 ± 0.004	2.120 ± 0.105
9	CFW-C10	*Bacillus cereus*	D	0.180 ± 0.231	2.972 ± 0.024	1.511 ± 0.046
10	CFW-C18	*Microbulbifer* sp.	D	0.329 ± 0.118	2.996 ± 0.193	2.439 ± 0.532
11	SBC-C23	*Bacillus* sp.	A	0.397 ± 0.047	3.131 ± 0.008	2.036 ± 0.114
12	SBC-C26	*Tenacibaculum ascidiaceicola*	A	0.017 ± 0.078	2.920 ± 0.020	1.419 ± 0.033
13	SBC-C30	*Bacillus* sp.	A	0.085 ± 0.076	2.924 ± 0.007	1.425 ± 0.012
14	CFW-C32	*Bacillus pumilus*	D	0.160 ± 0.056	3.136 ± 0.005	2.272 ± 0.055
15	SBC-C34	*Muricauda sp.*	A	0.327 ± 0.109	3.068 ± 0.003	1.733 ± 0.013
16	SBW-C35	*Bacillus sp.*	B	0.209 ± 0.144	3.056 ± 0.005	1.699 ± 0.103
17	SBW-C40	*Bacillus* sp.	B	0.597 ± 0.261	3.028 ± 0.024	1.632 ± 0.059
18	MW-C41	*Microbulbifer mangrovi*	C	0.250 ± 0.319	2.858 ± 0.038	1.323 ± 0.054
19	MW-C42	*Bacillus paralicheniformis*	C	0.120 ± 0.084	3.110 ± 0.014	2.432 ± 0.071
20	MW-C44	*Bacillus* sp.	C	0.017 ± 0.078	3.090 ± 0.016	2.514 ± 0.058
21	MW-C45	*Bacillus* sp.	C	0.065 ± 0.069	3.040 ± 0.023	2.658 ± 0.058
22	MW-C47	*Bacillus* sp.	C	0.017 ± 0.078	2.937 ± 0.045	2.867 ± 0.076
23	MW-C48	*Vibrio* sp.	C	0.554 ± 0.176	2.684 ± 0.101	3.216 ± 0.119
24	MW-C52	*Bacillus subtilis*	C	0.325 ± 0.125	3.072 ± 0.011	2.573 ± 0.031
25	MW-C54	*Vibrionaceae*	C	0.060 ± 0.061	2.961 ± 0.005	1.490 ± 0.121
26	MW-C56	*Bacillus altitudinis*	C	0.069 ± 0.074	2.933 ± 0.024	1.441 ± 0.040
27	MW-C57	*Bacillus* sp.	C	2.325 ± 0.061	3.123 ± 0.022	–
28	MW-C58	*Microbulbifer mangrovi*	C	0.046 ± 0.025	3.120 ± 0.017	2.223 ± 0.223
29	MW-C59	*Bacillus* sp.	C	0.017 ± 0.078	3.045 ± 0.029	1.676 ± 0.073
30	MW-C60	*Bacillus firmus*	C	0.265 ± 0.141	3.055 ± 0.022	1.698 ± 0.058
31	MW-C61	*Bacillus* sp.	C	0.265 ± 0.068	2.977 ± 0.192	2.675 ± 0.376
32	MW-C62	*Bacillus cereus*	C	0.042 ± 0.036	2.979 ± 0.031	1.527 ± 0.059
33	MW-C63	*Vibrionaceae bacterium*	C	0.027 ± 0.015	3.118 ± 0.017	2.380 ± 0.098
34	MW-C65	*Microbulbifer mangrovi*	C	0.132 ± 0.163	2.933 ± 0.036	1.443 ± 0.062
35	MW-C67	*Bacillus cereus*	C	0.386 ± 0.204	2.726 ± 0.008	1.145 ± 0.010
36	MW-C69	*Vibrionaceae bacterium*	C	0.020 ± 0.005	2.994 ± 0.012	1.555 ± 0.025
37	MW-C71	*Vibrionaceae bacterium*	C	0.364 ± 0.077	2.794 ± 0.048	1.234 ± 0.062
38	MW-C72	*Microbulbifer taiwanensis*	C	0.051 ± 0.049	2.916 ± 0.020	1.413 ± 0.033
39	MW-C73	*Bacillus* sp.	C	0.101 ± 0.103	3.112 ± 0.021	1.903 ± 0.091
40	MW-C74	*Vibrionaceae bacterium*	C	0.170 ± 0.193	2.729 ± 0.081	1.154 ± 0.098
41	MW-C76	*Microbulbifer* sp.	C	0.017 ± 0.078	2.964 ± 0.032	1.498 ± 0.062
42	MW-C77	*Bacillus altitudinis*	C	0.128 ± 0.079	3.034 ± 0.018	2.675 ± 0.043
43	MW-C78	*Bacillus* sp.	C	0.130 ± 0.125	2.975 ± 0.063	1.531 ± 0.132
44	MW-C79	*Bacillus subtilis*	C	0.195 ± 0.127	2.550 ± 0.129	3.361 ± 0.138
45	MW-C81	*Bacillus subtilis*	C	0.200 ± 0.019	3.134 ± 0.008	2.042 ± 0.073
46	MW-C83	*Bacillus cereus*	C	0.018 ± 0.001	3.086 ± 0.010	1.789 ± 0.033
47	MW-C86	*Bacillus* sp.	C	0.077 ± 0.085	2.637 ± 0.049	1.043 ± 0.053
48	MW-C88	*Bacillus* sp.	C	0.071 ± 0.039	2.911 ± 0.023	1.404 ± 0.037
49	MW-M4	*Bacillus* sp.	C	0.017 ± 0.078	2.648 ± 0.043	3.263 ± 0.048
50	MW-M5	*Exiguobacterium* sp.	C	0.017 ± 0.078	1.790 ± 0.533	3.951 ± 0.367
51	MW-M9	*Vibrionaceae bacterium*	C	0.017 ± 0.078	3.098 ± 0.017	2.482 ± 0.065
52	MW-M10	*Bacillus* sp.	C	0.028 ± 0.016	1.965 ± 0.793	3.752 ± 0.610
53	MW-M13	*Bacillus* sp.	C	0.049 ± 0.046	3.104 ± 0.039	2.323 ± 0.258
54	MW-M14	*Bacillus cereus*	C	0.017 ± 0.078	2.343 ± 0.445	3.505 ± 0.406
55	MW-M15	*Bacillus* sp.	C	0.053 ± 0.051	3.097 ± 0.047	2.343 ± 0.276
56	MW-M17	*Bacillus* sp.	C	0.017 ± 0.078	3.117 ± 0.026	2.346 ± 0.164
57	MW-M19	*Vibrio* sp.	C	0.018 ± 0.002	3.061 ± 0.086	2.436 ± 0.350
58	MW-M20	*Bacillus* sp.	C	0.907 ± 0.056	2.820 ± 0.222	2.990 ± 0.327

*A, B, C, and D represent four sampling sites, two shrimp culture bases in Haiwei town (A: 19°26′39.02″N, 108°50′11.23″E) and Huiwen town (B: 19°27′28″N, 110°45′13″E), seagrass beds around the mangrove in Huiwen town (C: 19°28′11.66″N, 110°47′41.22″E), and the seagrass beds in Dongjiao Coconut Forest, Huiwen town (D: 19°31′28.81″N, 110°52′0.45″E). The HC values of less than 1 are not shown, and the REC of those isolates are also not shown.*

### Small-Scale Laboratory Simulation Experiment

In this study, 28 cellulolytic bacterial strains with HC value greater than 2 were selected to evaluate the safety of cellulolytic bacteria and SB decomposition of cellulolytic bacteria in the small-scale laboratory simulation of shrimp aquaculture. As shown in the part of the safety test of Table 2, of the 28 test cellulolytic marine bacteria, only CFW-C9 and MW-C47 were harmless to shrimp as no mortality was observed by subjecting the test animal to a high density of the cellulolytic marine bacteria (10^–7^ CFU ml^–1^) for 1 week. In addition, the SB decomposition of the cellulolytic bacteria were also evaluated in the small-scale laboratory simulation of shrimp aquaculture. As shown in the part of SB decomposition test of Table 2, CFW-C18, MW-C44, MW-C77, and MW-M19 displayed SB decomposition rates of more than 60% after treatments for 1 week, and CFW-C18 exhibited the highest SB decomposition rate of 75.31% with the relatively high C/N ratio of 14.34, followed by MW-M19 of 74.35% with the relatively high C/N ratio of 14.75. Meanwhile, compared with the positive control group (containing SB; initial C/N ratio: approximately 12), the C/N ratios of the rearing water were increased after the treatments of CFW-C18, MW-C44, MW-C77, and MW-M19 due to the decrease in nitrogen content, whereas those were decreased after the treatments of other strains.

**TABLE 2:** The safety evaluation of cellulolytic bacteria and the SB decomposition of cellulolytic bacteria after rearing shrimps for 1 week in small-scale laboratory simulation.

	Safety test		SB decomposition test		
	Survival rate (%)	MAR index	SBase	C/N ratio	Decomposition rate (%)
CFW-C18	93.33 ± 11.55	0.47	2.36 ± 0.35	14.34 ± 2.36	75.31 ± 4.58
CFW-C32	85.00 ± 5.00	0.30	0.98 ± 0.26	8.23 ± 1.34	31.68 ± 5.67
CFW-C6	96.67 ± 5.77	0.70	1.79 ± 0.38	11.25 ± 1.08	40.35 ± 7.23
CFW-C7	93.33 ± 5.77	0.70	–	7.59 ± 1.38	20.56 ± 6.12
CFW-C9	0.00	0.43	–	7.35 ± 2.36	10.45 ± 3.56
MW-C42	100.00 ± 0.00	0.63	2.36 ± 0.37	10.36 ± 0.97	48.24 ± 5.38
MW-C44	90.00 ± 10.00	0.70	3.78 ± 0.42	13.62 ± 2.54	63.59 ± 7.44
MW-C45	90.00 ± 10.00	0.73	–	8.26 ± 1.95	34.61 ± 6.37
MW-C47	3.33 ± 5.77	0.67	–	7.98 ± 3.45	18.85 ± 4.99
MW-C48	93.33 ± 5.77	0.93	2.57 ± 0.82	11.33 ± 2.36	55.43 ± 7.31
MW-C52	86.67 ± 5.77	0.83	0.54 ± 0.30	7.46 ± 2.18	20.66 ± 6.83
MW-C58	93.33 ± 5.77	0.73	0.78 ± 0.48	7.25 ± 4.95	27.64 ± 8.34
MW-C61	96.67 ± 5.77	0.87	0.91 ± 0.15	7.56 ± 3.54	16.35 ± 5.75
MW-C63	93.33 ± 5.77	0.70	2.33 ± 0.54	10.44 ± 2.54	46.37 ± 4.98
MW-C77	86.87 ± 5.77	0.73	4.56 ± 0.44	13.32 ± 1.89	65.34 ± 5.89
MW-C79	93.33 ± 5.77	0.8	3.54 ± 0.56	12.85 ± 1.87	45.55 ± 8.35
MW-C81	100.00 ± 0.00	0.83	0.88 ± 0.31	8.36 ± 1.21	23.45 ± 6.87
MW-M10	93.33 ± 5.77	0.67	0.72 ± 0.35	7.97 ± 2.65	26.33 ± 7.23
MW-M13	96.67 ± 5.77	0.43	1.45 ± 0.04	10.33 ± 1.24	44.38 ± 3.25
MW-M14	90.00 ± 10.00	0.47	–	8.25 ± 2.47	12.34 ± 5.32
MW-M15	93.33 ± 5.77	0.23	2.35 ± 0.21	12.44 ± 1.04	58.36 ± 8.72
MW-M17	96.67 ± 5.77	0.40	2.28 ± 0.37	11.32 ± 1.35	42.34 ± 5.28
MW-M19	100.00 ± 0.00	0.10	5.34 ± 0.74	14.75 ± 2.48	74.35 ± 7.35
MW-M20	96.67 ± 5.77	0.73	2.24 ± 0.28	10.25 ± 3.42	49.98 ± 8.21
MW-M4	100.00 ± 0.00	0.77	–	7.96 ± 2.14	18.32 ± 4.37
MW-M5	86.67 ± 11.55	0.27	–	8.33 ± 2.16	10.37 ± 4.84
MW-M9	100.00 ± 0.00	0.37	–	7.01 ± 3.21	20.36 ± 4.78
SBC-C23	96.67 ± 5.77	0.57	–	8.03 ± 2.36	17.66 ± 7.29
Control-1	100.00 ± 0.00	–	–	7.25 ± 1.95	7.35 ± 4.95
Control-2	100.00 ± 0.00	–	–	8.54 ± 0.87	15.34 ± 4.35

*The experimental shrimp groups were immersed in bacterial suspension at a concentration of about 10^7^ CFU ml^–1^ for the evaluation of the safety of cellulolytic bacteria, and 10^5^ CFU ml^–1^ of rearing water for the evaluation of SB decomposition of cellulolytic bacteria. Control-1: shrimps were immersed in rearing water without the supplement of SB and bacteria. Control-2: shrimps were immersed in rearing water containing 4 g/L of SB powder without the supplement of bacteria. The initial C/N ratio of the experimental shrimp groups without SB were approximately 10, and those containing SB were approximately 12.*

### Antibiotic Susceptibility Assay

The antibiotic susceptibilities of 28 cellulolytic marine bacteria belonging to three genera (22 strains of *Bacillus*, 4 strains of *Vibrio*, and 1 strain of *Micrococcus*) were measured with 30 kinds of antibiotics (Table 3 and Supplementary Tables 3, 4). As shown in Table 3, 82.14% of the cellulolytic marine bacteria were susceptible to chloramphenicol. Around 50–80% of cellulolytic marine bacteria were intermediately resistant to tested antibiotics, such as cefoxitin (67.86%), ceftriaxone (78.57%), neomycin (53.57%), oxytetracycline (57.14%), and furazolidone (50%), and 80–93% of the cellulolytic marine bacteria were resistant to tested antibiotics, such as penicillin (89.29%), oxacillin (89.29%), piperacillin (89.29%), carbenicillin (82.14%), cefalexin (82.14%), streptomycin (85.71%), spectinomycin (82.14%), tetracycline (92.86%), erythromycin (85.71%), and azithromycin (85.71%). Also, 77.27% of *Bacillus* were sensitive to phenylpropanol chloramphenicol, and 100% of *Vibrio* and *Micrococcus* were sensitive to chloramphenicol (Table 3). The strains performed strong resistance to some antibiotics with higher MAR index, such as penicillin (0.89), piperacillin (0.89), carbenicillin (0.82), cefalexin (0.82), streptomycin (0.86), spectinomycin (0.82), tetracycline (0.93), erythromycin (0.86), and azithromycin (0.86). Antibiotic-resistant bacteria in mariculture farms may be transported by water current flowing from surrounding farms that utilized antibiotics excessively. These strains were not resistant to some antibiotics with lower MAR index, such as cefoxitin (0.18), ceftriaxone (0.14), chloramphenicol (0.04), and furazolidone (0.18). MW-M19 belonging to *Vibrio* sp. with the lowest MAR index (0.1) was resistant to only three antibiotics, while MW-C47 belonging to *Bacillus* with the highest MAR index (0.93) was resistant to 28 kinds of antibiotics (Table 3 and Supplementary Table 4).

**TABLE 3:** Resistance patterns of 28 cellulase-producing marine bacteria according to antimicrobial susceptibility test using 30 kinds of antibiotics.

Antibiotic tested		Cellulolytic marine bacteria (%)			Susceptibility in genus level (%)			MAR index
		S	I	R	*Bacillus*	*Vibrio*	*Micrococcus*	
Penicillin	PEN	7.14%	3.57%	89.29%	4.55%	25.00%	0.00%	0.89
Ampicillin	AMP	17.86%	17.86%	64.29%	13.64%	50.00%	0.00%	0.64
Amoxicillin	AMX	35.71%	3.57%	60.71%	31.82%	50.00%	50.00%	0.61
Oxacillin	OXA	10.71%	0.00%	89.29%	4.55%	50.00%	0.00%	0.89
Piperacillin	PIP	10.71%	0.00%	89.29%	9.09%	25.00%	0.00%	0.89
Carbenicillin	CAR	10.71%	7.14%	82.14%	9.09%	25.00%	0.00%	0.82
Cefazolin	CFZ	17.86%	21.43%	60.71%	13.64%	50.00%	0.00%	0.61
Cefalexin	LEX	10.71%	7.14%	82.14%	9.09%	25.00%	0.00%	0.82
Cefoxitin	FOX	14.29%	67.86%	17.86%	9.09%	50.00%	0.00%	0.18
Ceftriaxone	CRO	7.14%	78.57%	14.29%	0.00%	25.00%	50.00%	0.14
Streptomycin	STR	7.14%	7.14%	85.71%	4.55%	0.00%	50.00%	0.86
Kanamycin	KAN	39.29%	3.57%	57.14%	0.00%	0.00%	50.00%	0.57
Gentamicin	GEN	17.86%	25.00%	57.14%	13.64%	25.00%	50.00%	0.57
Amikacin	AMK	3.57%	21.43%	75.00%	0.00%	0.00%	50.00%	0.75
Neomycin	NEO	7.14%	53.57%	39.29%	0.00%	25.00%	50.00%	0.39
Spectinomycin	SPE	7.14%	10.71%	82.14%	0.00%	25.00%	50.00%	0.82
Oxytetracycline	OXY	3.57%	57.14%	39.29%	0.00%	0.00%	50.00%	0.39
Tetracycline	TCY	3.57%	3.57%	92.86%	0.00%	0.00%	50.00%	0.93
Minocycline	MNO	14.29%	21.43%	64.29%	9.09%	25.00%	50.00%	0.64
Doxycycline	DOX	17.86%	10.71%	71.43%	9.09%	50.00%	50.00%	0.71
Florfenicol	FFC	17.86%	32.14%	50.00%	9.09%	50.00%	50.00%	0.50
Chloramphenicol	CHL	82.14%	14.29%	3.57%	77.27%	100.00%	100.00%	0.04
Furazolidone	FRZ3	32.14%	50.00%	17.86%	31.82%	50.00%	0.00%	0.18
Norfloxacin	NOR	28.57%	46.43%	25.00%	27.27%	25.00%	50.00%	0.25
Levofloxacin	LVX	14.29%	28.57%	57.14%	13.64%	0.00%	50.00%	0.57
Ofloxacin	OFX	28.57%	21.43%	50.00%	27.27%	25.00%	50.00%	0.50
Ciprofloxacin	CIP	14.29%	35.71%	50.00%	9.09%	25.00%	50.00%	0.50
Erythromycin	ERY	7.14%	7.14%	85.71%	0.00%	25.00%	50.00%	0.86
Azithromycin	AZM	7.14%	7.14%	85.71%	0.00%	25.00%	50.00%	0.86
Rifampicin	RIF	14.29%	14.29%	71.43%	13.64%	0.00%	50.00%	0.71

*S, sensitive to tested antibiotic; I, intermediate resistant to tested antibiotic; R, resistant to tested antibiotic.*

## Discussion

The South China Sea (SCS), as one of the largest marginal seas with rich microbial resources, covers more than 2 million km^2^, which has a lot of seagrass ecosystems considered as major blue carbon sinks with high microbial diversity (Pendleton et al., 2012; Lavery et al., 2013; Xu et al., 2017; Gullström et al., 2018). We reason that bacteria with high cellulolytic activates are likely to exist in these seagrass ecosystems. Additionally, although high-density aquaculture can bring high profits, the accumulation of high-protein bait and excrement will aggravate the deterioration of the aquaculture water (Li et al., 2020). Residual bait and feces are the main sources of nitrogen and phosphorus during the processing of aquaculture, while the relative lack of carbon will lead to water deterioration (Lin and Chen, 2001, 2003; Hoang et al., 2020; Mariane de Morais et al., 2020; Penalosa-Martinell et al., 2020). However, the high cost of adding a carbon source artificially limits its application of large-scale popularization. Therefore, it has become a research hotspot to find cheap carbon sources for the cost reduction of aquaculture. Sugarcane is a frequently grown sugar crop in Hainan Island, China, and its SB is the main waste of the sugar industry. Normally, one ton of sugarcane produces 280 kg of SB, which is rich in fermentable components and usually composed of 25% lignin, 25% hemicelluloses, and 50% cellulose (Soccol et al., 2010; Ejaz et al., 2019). However, there was no report on SB decomposition of cellulolytic marine bacterial strains for the application of shrimp aquaculture in Hainan Island. Therefore, our aim of this work is to isolate cellulolytic marine bacteria from these areas to reduce aquaculture cost by hydrolyzing SB that is characterized by cheap and easily available carbon source in Hainan Island located in SCS. Interestingly, 91 cellulolytic marine bacteria (dominated by *Bacillus*) of 97 cellulolytic marine isolates were from seagrass bed ecosystems of Hainan Island, whereas only six cellulolytic bacterial strains were from the aquaculture environments (Supplementary Table 1), indicating that seagrass beds can be used as an important place for screening cellulolytic bacteria. Reportedly, the cellulolytic bacteria isolated from different areas displayed different properties, e.g., the cellulolytic bacterium from a farm at Zhanjiang (Guangdong Province, China), *Lactobacillus pentosus* AS13, can effectively enhance the growth performance, feed utilization, digestive enzymes, and disease resistance of *L. vannamei* (Zheng and Wang, 2017); *Geobacillus thermodenitrificans* IP_WH1 from North West Himalayas could produce thermotolerant cellulase (Priya et al., 2016). Herein, we isolated a lot of tropical cellulolytic bacteria with excellent SB decomposition, especially CFW-C18, MW-C44, MW-C77, and MW-M19 that displayed the SB decomposition rate of more than 60% after treatments for 1 week in a small-scale laboratory simulation of shrimp aquaculture.

Currently, various cellulolytic microorganisms were from fungi, such as *Aspergillus* (Mohapatra et al., 2018), *Penicillium* (Han et al., 2017), *Trichoderma* (Wang et al., 2020), and *Talaromyces* (Liao et al., 2018), etc., which required long production cycles. Compared with fungi, bacteria not only have a higher growth rate but also produce cellulase in a short time. At the present study, 58 bacterial strains with HC values of more than 1 were characterized by the secretion of cellulase, of which 28 cellulolytic marine bacterial strains displayed the HC values of more than 2 (Table 1), which were very similar to that from mangrove soils (HC values ranged from 1.25 to 2.5) (Behera et al., 2014), flower stalk–vegetable waste co-composting system (HC values ranging from 0.4 to 2.1) (Lu et al., 2005), and oil palm (HC values ranging from 1.56 to 4.14) (Khianngam et al., 2014). Additionally, the FPase activities of these marine bacteria, ranging from 0.018 to 2.325 U/ml from these study areas, were similar to that from Haryana (Saini et al., 2017). It is worth noting that there were 30 cellulolytic marine strains with a relative enzyme content of more than 3 U/g in the supernatant of fermentation broth after 24 h according to the linear relationship between HC value and content of commercial cellulase, indicating that this method for quantitatively analyzing the detected enzymic production of the strains according to the standard curve of known enzyme is more intuitive, faster, and more effective, which will provide a new idea for the method of measuring enzymic activity of cellulolytic strains.

People usually use probiotics to replace antibiotics in the process of aquaculture of aquatic animals, which cannot only restrain the multiplying of pathogen and enhance the immunity of cultured animals but also improve the aquaculture environment (Peñalosa-Martinell et al., 2020; Ren et al., 2021). However, in order to be considered as a probiotic, the strain has to be non-toxic to the host. Therefore, the 28 cellulolytic bacterial strains with an HC value of greater than 2 were selected to evaluate the safety of cellulolytic bacteria in a small-scale laboratory simulation of shrimp aquaculture, of which 26 test cellulolytic marine bacteria were safe for *L. vannamei* (Table 2). In addition to the detection of animal safety of these cellulolytic bacteria, antibiotic susceptibility analysis is also an effective method for identifying potential probiotics. The abuse of antibiotics will destroy the balance of the original microbial flora in the breeding environment, resulting in drug resistance. At the present study, the main purpose was to screen the highly safe strains with low drug resistance that are suitable for the application in shrimp aquaculture to reduce costs by the supplement of SB. Most test cellulolytic marine bacteria were susceptible to chloramphenicol. According to our results, the animals in the study environments had been threatened by penicillin, piperacillin, carbenicillin, cefalexin, streptomycin, spectinomycin, tetracycline, erythromycin, and azithromycin, while some antibiotics with relatively lower MAR index could be reasonably used in these environments, such as cefoxitin, ceftriaxone, and furazolidone. More importantly, WM-M19 displayed a lowest MAR index (0.1), which was resistant to only three antibiotics, including streptomycin, amikacin, and levofloxacin, indicating that this strain was safe and non-drug resistant for further use. It is worth noting that CFW-C18 with the highest SB decomposition (75.31%) also displayed a relatively low MAR index (0.47). Of course, antibiotic resistance pattern may vary depending on the geographical locations and selective pressure, and these patterns change rapidly from time to time (Kathleen et al., 2016).

Meanwhile, 28 cellulolytic bacterial strains with an HC value greater than 2 were also selected to evaluate the SB decomposition of cellulolytic bacteria in a small-scale laboratory simulation of shrimp aquaculture for the demonstration of the capability of the isolated bacteria under the conditions relevant to the target application. CFW-C18 and MW-M19 exhibited excellent SB decomposition capabilities, and the SB decomposition rates of both were up to 75.31 and 74.35%, respectively, which were similar or higher than the result of *in vitro* (Supplementary Table 5; the details are presented in the Supplementary Material) and those reported previously, such as the three fungi, *Phanerochaete chrysosporium* PC2, *Lentinula edode* LE16, and *Pleurotus ostreatus* PO45, which removed 73.5, 45.5, and 32.2% of total SB in 12 weeks, respectively, in which *P. chrysosporium* PC2 was the most efficient strain to degrade all the ingredients of SB (Dong et al., 2013); two fungi, *Cellulomonas* sp. and *Aspergillus terreus*, displayed high cellulose consumptions in bagasse alkali treated at high temperatures, up to 90 and 72.5%, respectively (Garg and Neelakantan, 1982); the bacterial consortium of MC3F-phylotypes degraded 59.4 ± 2.2% of alkali–peracetic acid-treated SB in 1 week (Wongwilaiwalin et al., 2010), and the saccharification of bacteria-treated SB, *Bacillus subtilis* CD001, was up to 39.21% after 30 days (Malik et al., 2021).

Additionally, compared with the initial C/N ratio of the positive control group (containing SB; initial C/N ratio approximately 12), the final C/N ratios of the rearing water after the treatments of cellulolytic bacteria except CFW-C18, MW-C44, MW-C77, and MW-M19, were decreased, whereas those compared with the final C/N ratios of the rearing water of the negative group (Table 2; without supplement of cellulolytic bacteria and SB; final C/N ratio was approximately 7) were increased, indicating that the supplement of these cellulolytic bacteria and SB could alleviate the decrease in C/N ratio, and even improve the C/N ratio of rearing water such as CFW-C18, MW-C44, MW-C77, and MW-M19, especially CFW-C18 (C/N ratio of 14.34) and MW-M19 (C/N ratio of 14.75).

Overall, *Microbulbifer* sp. CFW-C18 and *Vibrio* sp. MW-M19 can be considered as the ideal cellulolytic marine strains for the potential utilization of SB as a low-cost carbon source in shrimp aquaculture, since both of them were characterized by high SB decomposition, low drug resistance (MAR index), and safe for shrimps, which will lay a foundation for the development of cellulase-producing marine microbial agents for shrimp aquaculture based on the artificial addition of SB as a carbon source.

## Conclusion

Sugarcane bagasse characterized by cheap and easily available carbon source is widely distributed in Hainan Island. In this work, we reported 97 tropical cellulolytic bacteria isolated from Hainan Island for the potential utilization of SB as a low-cost carbon source in shrimp aquaculture, of which 91 cellulolytic bacteria were from seagrass beds. We reason that the seagrass beds are of immense importance for the isolation of cellulolytic bacteria. More importantly, we successfully obtained and identified two cellulolytic marine bacteria, *Microbulbifer* sp. CFW-C18 and *Vibrio* sp. MW-M19, and both of them besides characterized by excellent SB decomposition, low drug resistance (MAR index), and safe for shrimps, also increased the C/N ratios by decreasing the nitrogen content after the supplement of SB, which will make them proficient candidates for shrimp aquaculture based on low-cost external carbon sources and even for other biotechnological applications.

## Data Availability Statement

The original contributions presented in the study are included in the article/Supplementary Material, further inquiries can be directed to the corresponding author.

## Author Contributions

WR and ZX designed the study, wrote the manuscript, and analyzed the data. XX, HL, XZ, XC, and AH performed the experiments. All authors contributed to the article and approved the submitted version.

## Conflict of Interest

The authors declare that the research was conducted in the absence of any commercial or financial relationships that could be construed as a potential conflict of interest.

## Publisher’s Note

All claims expressed in this article are solely those of the authors and do not necessarily represent those of their affiliated organizations, or those of the publisher, the editors and the reviewers. Any product that may be evaluated in this article, or claim that may be made by its manufacturer, is not guaranteed or endorsed by the publisher.

## Footnotes

1
https://eztaxon-e.ezbiocloud.net/
