# Supplementary material for: Tropical Cellulolytic Bacteria: Potential Utilization of Sugarcane Bagasse as Low-Cost Carbon Source in Aquaculture
Source: Front Microbiol. 2021 Oct 29;12:745853. doi: 10.3389/fmicb.2021.745853 (PMC8586208; doi:10.3389/fmicb.2021.745853)
Supplement: Supplementary file 1 [file Data_Sheet_1.docx]

Tropical cellulolytic bacteria: Potential utilization of sugarcane bagasse as low-cost carbon source in aquaculture

**Wei Ren^1, 2, 3, 4^,** **Xueni Xu^2, 3^, Hao Long^1, 2, 3, 4^, Xiang Zhang^1, 2, 3, 4^, Xiaoni Cai^1, 2, 3, 4^, Aiyou Huang^1, 2, 3, 4^, Zhenyu Xie^1, 2, 3, 4^***

^1^ State Key Laboratory of Marine Resource Utilization in the South China Sea, Hainan University, Haikou, 570228, Hainan Province, China.

^2^ Hainan Provincial Key Laboratory for Tropical Hydrobiology and Biotechnology, Hainan University, Haikou, 570228, Hainan Province, China.

^3^ College of Marine Sciences, Hainan University, Haikou, 570228, Hainan Province, China.

^4^ Laboratory of Development and Utilization of Marine Microbial Resource, Hainan University, Haikou, 570228, Hainan Province, China.

*** Correspondence:**Corresponding Author

xiezyscuta@163.com (Zhenyu Xie)

.

**Isolation and purification of marine bacteria**

The samples were serially diluted with sterile normal saline solution (0.85% NaCl) within 24 h of collection to obtain 1:10, 1:10, 1:100, and 1:1,000 dilutions. One hundred microlitres of each diluted sample was spread-plated on marine 2216E agar and incubated at 30°C for 18 h. The agar plates were investigated in terms of colony morphology including shape, margin, elevation and pigmentation. Morphologically dissimilar colonies were selected and streak plated on marine 2216E agar to obtain pure colonies.

A total of 2,585 marine bacteria with dissimilarly morphological colonies were isolated from four sites in Hainan (Table S1). Among them, ninety-seven bacterial isolates were defined as cellulolytic bacteria because they exhibited the cellulolytic zone around their colonies on 2661E agar after Congo red staining, which were belonged to 6 genera (Figure S1 and Table S2). The largest genus was *Bacillus* (64%), followed by *Vibrio* (18%). The remaining genera were shared by *Microbulbifer* (10%), *Pseudomonas* (6%), *Tenacibaculum* (1%), and *Muricauda* (1%). We found that the cellulolytic bacteria were mainly from the natural environments (seagrass beds of Mangrove and Dongjiao Coconut Forest). However, there were only 6 cellulolytic bacteria isolated from aquaculture environments (two shrimp cultural bases). As shown in Fig. 1, *Bacillus* was accounted for the most in natural environment and aquaculture environment, followed by *Vibrio*. Interestingly, *Vibrio*, *Pseudomonas*, and *Microbulbifer* were only isolated from the natural environment, while *Tenacibaculum* and *Muricauda* were only isolated from the aquaculture environment.

**Table S1.** Marine bacterial strains isolated from four sites.

|  | Sampling site | Sample type | Total number of bacteria | Number of bacteria with  HC |
| --- | --- | --- | --- | --- |
| Aquaculture | shrimp cultural base in Haiwei Town (19°26′39.02″N, 108°50′11.23′′E) | Shrimp, water, mud | 335 | 4 |
|  | shrimp cultural base in Huiwen Town  (19°27′28″N, 110°45′13″E) | Shrimp, water, mud | 658 | 2 |
| Nature | Mangrove in Huiwen Town | mud | 1202 | 58 |
|  | Dongjiao Coconut Forest in Huiwen Town | mud | 390 | 33 |

**Figure S1 (A)** Congo red staining of standard commercial enzymes at different dilution concentrations. Note: 1: 0.0 mg/mL; 2: 0.05 mg/mL; 3: 0.025 mg/mL; 4: 0.0125 mg/mL; 5: 0.00625 mg/mL; 6: 0.003125 mg/mL; 7: 0.0015625 mg/mL; 8: 0.00078125 mg/mL. (B) Standard commercial enzyme standard curve.


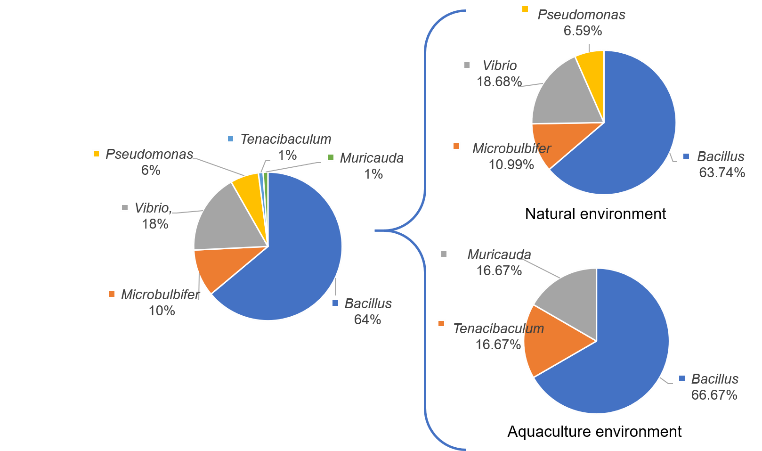


**Figure S2** Diversity of 97 cellulolytic marine bacteria in the study sites.

**Table S2.** The information of the antibiotic used in this study.

| Antibiotic Classes | Antibiotic tested | Disc content | Abbreviation | Susceptible (mm) | Intermediary (mm) | Resistant (mm) |
| --- | --- | --- | --- | --- | --- | --- |
| Penicillins | Penicillin | 10 μg | PEN | ≤19 |  | ≥20 |
|  | Ampicillin | 10 μg | AMP | ≤13 | 14-16 | ≥17 |
|  | Amoxicillin | 10 μg | AMX | ≤13 | 14-17 | ≥18 |
|  | Oxacillin | 1 μg | OXA | ≤13 | 14-17 | ≥18 |
|  | Piperacillin | 100 μg | PIP | ≤17 | 18-20 | ≥21 |
|  | Carbenicillin | 100 μg | CAR | ≤19 | 20-22 | ≥23 |
| Cephalosporins | Cefazolin | 30 μg | CFZ | ≤14 | 15-17 | ≥18 |
|  | Cefalexin | 30 μg | LEX | ≤14 | 15-17 | ≥18 |
|  | Cefoxitin | 30 μg | FOX | ≤14 | 15-17 | ≥18 |
|  | Ceftriaxone | 30 μg | CRO | ≤13 | 14-20 | ≥21 |
| Aminoglycosides | Streptomycin | 10 μg | STR | ≤11 | 12-14 | ≥15 |
|  | Kanamycin | 30 μg | KAN | ≤13 | 14-17 | ≥18 |
|  | Gentamicin | 10 μg | GEN | ≤12 | 13-14 | ≥15 |
|  | Amikacin | 30 μg | AMK | ≤14 | 15-16 | ≥17 |
|  | Neomycin | 30 μg | NEO | ≤12 | 13-16 | ≥17 |
| Aminocyclitols | Spectinomycin | 100 μg | SPE | ≤14 | 15-17 | ≥18 |
| Tetracyclines | Oxytetracycline | 30 μg | OXY | ≤4 | 4-16 | ≥17 |
|  | Tetracycline | 30 μg | TCY | ≤14 | 15-18 | ≥19 |
|  | Minocycline | 30 μg | MNO | ≤14 | 15-18 | ≥19 |
|  | Doxycycline | 30 μg | DOX | ≤12 | 13-15 | ≥16 |
| Amphenicols | Florfenicol | 30 μg | FFC | ≤25 | 26-28 | ≥29 |
|  | Chloramphenicol | 30 μg | CHL | ≤12 | 13-17 | ≥18 |
| Nitrofurans | Furazolidone | 300 μg | FRZ3 | ≤14 | 15-16 | ≥17 |
| Fluoroquinolones | Norfloxacin | 10 μg | NOR | ≤12 | 13-16 | ≥17 |
|  | Levofloxacin | 5 μg | LVX | ≤13 | 14-16 | ≥17 |
|  | Ofloxacin | 5 μg | OFX | ≤12 | 13-15 | ≥16 |
|  | Ciprofloxacin | 5 μg | CIP | ≤15 | 16-20 | ≥21 |
| Macrolides | Erythromycin | 15 μg | ERY | ≤13 | 14-22 | ≥23 |
|  | Azithromycin | 15 μg | AZM | ≤13 | 14-17 | ≥18 |
| Ansamycins | Rifampicin | 5 μg | RIF | ≤16 | 17-19 | ≥20 |

**Table S3.** Colony morphology and Fpase of 97 cellulolytic bacteria.

| Isolates code and species | | | site | Shape | Pigmentation | Fpase (U/mL) |
| --- | --- | --- | --- | --- | --- | --- |
| 1 | MW-C2 | *Bacillus aquimaris* | D | Circular | Yellow | 0.017 ± 0.078 |
| 2 | MW-C3 | *Microbulbifer* sp. | D | Circular | Pale yellow | 0.090 ± 0.104 |
| 3 | MW-C4 | *Microbulbifer* sp. | D | Circular | Pale yellow | 0.196 ± 0.244 |
| 4 | CFW-C5 | *Bacillus* sp. | D | Circular | White | 0.017 ± 0.078 |
| 5 | CFW-C6 | *Bacillus safensis* sp. | D | Circular | Pale yellow | 0.288 ± 0.097 |
| 6 | CFW-C7 | *Bacillus subtilis* | D | Circular | White | 0.342 ± 0.056 |
| 7 | CFW-C8 | *Bacillus* sp. | D | Circular | White | 0.017 ± 0.078 |
| 8 | CFW-C9 | *Bacillus sp.* | D | Circular | White | 0.145 ± 0.003 |
| 9 | CFW-C10 | *Bacillus cereus* | D | Circular | White | 0.180 ± 0.231 |
| 10 | CFW-C11 | *Vibrio alginolyticus* | D | Circular | White | 0.017 ± 0.078 |
| 11 | CFW-C12 | *Vibrio sp.* | D | Circular | White | 0.172 ± 0.083 |
| 12 | CFW-C13 | *Bacillus subtilis* | D | Circular | White | 0.196 ± 0.071 |
| 13 | CFW-C14 | *Vibrio alginolyticus* | D | Circular | White | 0.111 ± 0.067 |
| 14 | CFW-C15 | *Pseudomonas stutzeri* | D | Circular | White | 0.211 ± 0.247 |
| 15 | CFW-C16 | *Vibrio alginolyticus* | D | Circular | White | 0.025 ± 0.012 |
| 16 | CFW-C17 | *Bacillus subtilis* | D | Circular | White | 0.428 ± 0.110 |
| 17 | CFW-C18 | *Microbulbifer* sp. | D | Irregular | White | 0.329 ± 0.118 |
| 18 | CFW-C19 | *Microbulbifer* sp. | D | Circular | Colorless | 0.043 ± 0.037 |
| 19 | CFW-C20 | *Microbulbifer variabilis* | D | Circular | White | 0.017 ± 0.078 |
| 20 | CFW-C21 | *Pseudomonas stutzeri* | D | Circular | White | 0.022 ± 0.007 |
| 21 | CFW-C22 | *Vibrio* sp. | D | Circular | White | 0.017 ± 0.078 |
| 22 | SBC-C23 | *Bacillus* sp. | A | Circular | White | 0.397 ± 0.047 |
| 23 | CFW-C24 | *Bacillus subtilis* | D | Circular | White | 0.017 ± 0.078 |
| 24 | CFW-C25 | *Vibrio alginolyticus* | D | Circular | White | 0.118 ± 0.075 |
| 25 | SBC-C26 | *Tenacibaculum ascidiaceicola* | A | Circular | White | 0.017 ± 0.078 |
| 26 | CFW-C27 | *Pseudoalteromonas* sp. | D | Circular | Pale yellow | 0.017 ± 0.078 |
| 27 | CFW-C28 | *Bacillus* sp. | D | Circular | White | 0.140 ± 0.175 |
| 28 | CFW-C29 | *Bacillus* sp. | D | Circular | Pale yellow | 0.100 ± 0.119 |
| 29 | SBC-C30 | *Bacillus* sp. | A | Circular | White | 0.085 ± 0.076 |
| 30 | CFW-C31 | *Bacillus pumilus* | D | Circular | White | 0.019 ± 0.004 |
| 31 | CFW-C32 | *Bacillus pumilus* | D | Circular | White | 0.160 ± 0.056 |
| 32 | CFW-C33 | *Pseudoalteromonas* sp. | D | Circular | White | 0.017 ± 0.078 |
| 33 | SBC-C34 | *Muricauda sp.* | A | Irregular | White | 0.327 ± 0.109 |
| 34 | SBW-C35 | *Bacillus sp.* | B | Irregular | White | 0.209 ± 0.144 |
| 35 | CFW-C36 | *Bacillus pumilus* | D | Circular | White | 0.017 ± 0.078 |
| 36 | CFW-C37 | *Bacillus pumilus* | D | Circular | Pale yellow | 0.085 ± 0.053 |
| 37 | CFW-C38 | *Pseudoalteromonas* sp. | D | Circular | Pale yellow | 0.350 ± 0.150 |
| 38 | CFW-C39 | *Pseudoalteromonas* sp. | D | Circular | Pale yellow | 0.017 ± 0.078 |
| 39 | SBW-C40 | *Bacillus* sp. | B | Circular | Yellow | 0.597 ± 0.261 |
| 40 | MW-C41 | *Microbulbifer mangrovi* | C | Circular | White | 0.250 ± 0.319 |
| 41 | MW-C42 | *Bacillus paralicheniformis* | C | Circular | White | 0.120 ± 0.084 |
| 42 | MW-C43 | *Vibrio alginolyticus* | C | Circular | White | 0.017 ± 0.078 |
| 43 | MW-C44 | *Bacillus* sp. | C | Circular | White | 0.017 ± 0.078 |
| 44 | MW-C45 | *Bacillus* sp. | C | Circular | White | 0.065 ± 0.069 |
| 45 | MW-C46 | *Bacillus* sp. | C | Circular | White | 0.017 ± 0.078 |
| 46 | MW-C47 | *Bacillus* sp. | C | Circular | Yellow | 0.017 ± 0.078 |
| 47 | MW-C48 | *Vibrio* sp. | C | Circular | Yellow | 0.554 ± 0.176 |
| 48 | MW-C49 | *Bacillus* sp. | C | Circular | White | 0.053 ± 0.028 |
| 49 | MW-C50 | *Bacillus* sp. | C | Circular | Colorless | 0.293 ± 0.224 |
| 50 | MW-C51 | *Bacillus* sp. | C | Circular | White | 0.017 ± 0.078 |
| 51 | MW-C52 | *Bacillus subtilis* | C | Circular | Pale yellow | 0.325 ± 0.125 |
| 52 | MW-C53 | *Bacillus subtilis* | C | Circular | White | 0.019 ± 0.004 |
| 53 | MW-C54 | *Vibrionaceae bacterium* | C | Irregular | Pale yellow | 0.060 ± 0.061 |
| 54 | MW-C55 | *Bacillus cereus* | C | Irregular | White | 0.046 ± 0.037 |
| 55 | MW-C56 | *Bacillus altitudinis* | C | Circular | White | 0.069 ± 0.074 |
| 56 | MW-C57 | *Bacillus* sp. | C | Circular | White | 2.325 ± 0.061 |
| 57 | MW-C58 | *Microbulbifer mangrovi* | C | Oval | White | 0.046 ± 0.025 |
| 58 | MW-C59 | *Bacillus* sp. | C | Circular | White | 0.017 ± 0.078 |
| 59 | MW-C60 | *Bacillus firmus* | C | Oval | White | 0.265 ± 0.141 |
| 60 | MW-C61 | *Bacillus* sp. | C | Irregular | White | 0.265 ± 0.068 |
| 61 | MW-C62 | *Bacillus cereus* | C | Circular | White | 0.042 ± 0.036 |
| 62 | MW-C63 | *Vibrionaceae bacterium* | C | Circular | White | 0.027 ± 0.015 |
| 63 | MW-C64 | *Bacillus* sp. | C | Circular | White | 0.017 ± 0.078 |
| 64 | MW-C65 | *Microbulbifer mangrovi* | C | Circular | White | 0.132 ± 0.163 |
| 65 | MW-C66 | *Bacillus* sp. | C | Circular | Orange | 0.095 ± 0.111 |
| 66 | MW-C67 | *Bacillus cereus* | C | Circular | White | 0.386 ± 0.204 |
| 67 | MW-C68 | *Vibrionaceae bacterium* | C | Circular | Pale red | 0.017 ± 0.078 |
| 68 | MW-C69 | *Vibrionaceae bacterium* | C | Circular | White | 0.020 ± 0.005 |
| 69 | MW-C70 | *Bacillus* sp. | C | Oval | Pale white | 0.323 ± 0.051 |
| 70 | MW-C71 | *Vibrionaceae bacterium* | C | Circular | White | 0.364 ± 0.077 |
| 71 | MW-C72 | *Microbulbifer taiwanensis* | C | Circular | White | 0.051 ± 0.049 |
| 72 | MW-C73 | *Bacillus* sp. | C | Irregular | Pale yellow | 0.101 ± 0.103 |
| 73 | MW-C74 | *Vibrionaceae bacterium* | C | Circular | Pale yellow | 0.170 ± 0.193 |
| 74 | MW-C75 | *Bacillus altitudinis* | C | Circular | White | 0.017 ± 0.078 |
| 75 | MW-C76 | *Microbulbifer* sp. | C | Circular | White | 0.017 ± 0.078 |
| 76 | MW-C77 | *Bacillus altitudinis* | C | Circular | White | 0.128 ± 0.079 |
| 77 | MW-C78 | *Bacillus* sp. | C | Circular | Pale yellow | 0.130 ± 0.125 |
| 78 | MW-C79 | *Bacillus subtilis* | C | Circular | White | 0.195 ± 0.127 |
| 79 | MW-C80 | *Bacillus hwajinpoensis* | C | Irregular | White | 0.209 ± 0.194 |
| 80 | MW-C81 | *Bacillus subtilis* | C | Circular | White | 0.200 ± 0.019 |
| 81 | MW-C82 | *Vibrio* sp. | C | Circular | White | 0.121 ± 0.136 |
| 82 | MW-C83 | *Bacillus cereus* | C | Circular | White | 0.018 ± 0.001 |
| 83 | MW-C84 | *Bacillus* sp. | C | Irregular | Orange | 0.017 ± 0.078 |
| 84 | MW-C85 | *Bacillus cereus* | C | Circular | White | 0.205 ± 0.083 |
| 85 | MW-C86 | *Bacillus* sp. | C | Circular | Orange | 0.077 ± 0.085 |
| 86 | MW-C87 | *Bacillus* sp. | C | Circular | Yellow | 0.093 ± 0.047 |
| 87 | MW-C88 | *Bacillus* sp. | C | Circular | Pale yellow | 0.071 ± 0.039 |
| 88 | MW-M4 | *Bacillus* sp. | C | Circular | White | 0.017 ± 0.078 |
| 89 | MW-M5 | *Exiguobacterium* sp. | C | Circular | Pale yellow | 0.017 ± 0.078 |
| 90 | MW-M9 | *Vibrionaceae bacterium* | C | Circular | Pale yellow | 0.017 ± 0.078 |
| 91 | MW-M10 | *Bacillus* sp. | C | Circular | Pale yellow | 0.028 ± 0.016 |
| 92 | MW-M13 | *Bacillus* sp. | C | Circular | Pale yellow | 0.049 ± 0.046 |
| 93 | MW-M14 | *Bacillus cereus* | C | Circular | Pale yellow | 0.017 ± 0.078 |
| 94 | MW-M15 | *Bacillus* sp. | C | Circular | Pale yellow | 0.053 ± 0.051 |
| 95 | MW-M17 | *Bacillus* sp. | C | Circular | Pale yellow | 0.017 ± 0.078 |
| 96 | MW-M19 | *Vibrio* sp. | C | Circular | Pale yellow | 0.018 ± 0.002 |
| 97 | MW-M20 | *Bacillus* sp. | C | Circular | Pale yellow | 0.907 ± 0.056 |

Note: A, B, C, and D represent four sampling sites, two shrimp cultural bases in Haiwei Town (A: 19°26′39.02″N, 108°50′11.23′′E) and Huiwen Town (B: 19°27′28″N, 110°45′13″E), seagrass beds around mangrove in Huiwen Town (C: 19°28′11.66″N, 110°47′41.22′′E), and seagrass beds in Dongjiao Coconut Forest, Huiwen Town (D: 19°31′28.81″N, 110°52′0.45″E).

**Table S4.** Resistance patterns of 28 cellulase-producing marine bacteria according to antimicrobial susceptibility test using 30 kinds of antibiotics.

|  | PEN | AMP | AMX | OXA | PIP | CAR | CFZ | LEX | FOX | CRO | STR | KAN | GEN | AMK | NEO | SPE | OXY | TCY | MNO | DOX | FFC | CHL | FRZ3 | NOR | LVX | OFX | CIP | ERY | AZM | RIF |
| --- | --- | --- | --- | --- | --- | --- | --- | --- | --- | --- | --- | --- | --- | --- | --- | --- | --- | --- | --- | --- | --- | --- | --- | --- | --- | --- | --- | --- | --- | --- |
| CFW-C18 | R | R | S | R | R | R | R | R | R | S | S | S | S | S | S | S | S | S | S | S | S | S | R | S | S | S | S | S | S | S |
| CFW-C32 | R | R | R | R | R | R | R | R | I | I | R | R | I | R | I | R | I | R | R | R | I | S | I | I | I | R | R | R | R | I |
| CFW-C6 | R | I | I | R | R | R | I | R | I | I | I | I | I | I | R | I | I | R | R | R | R | S | S | S | S | S | I | R | R | R |
| CFW-C7 | R | I | S | R | R | R | I | R | I | I | I | I | S | R | I | R | R | R | R | R | I | S | I | S | S | S | S | R | R | R |
| CFW-C9 | R | R | R | R | R | R | R | R | I | I | R | R | I | R | I | R | I | R | R | R | I | S | I | S | I | I | I | I | R | R |
| MW-C42 | R | R | R | R | R | R | R | R | I | I | R | R | R | R | I | R | I | R | R | R | I | S | I | I | R | R | R | R | R | S |
| MW-C44 | R | R | R | R | R | R | R | R | I | I | R | R | R | R | I | R | I | R | I | R | R | I | I | I | R | R | R | R | R | R |
| MW-C45 | R | R | R | R | R | R | R | R | I | I | R | R | R | I | I | R | I | R | I | R | I | S | S | I | R | R | R | R | R | R |
| MW-C47 | R | R | R | R | R | R | R | R | R | R | R | R | R | R | R | R | R | R | R | R | R | I | R | R | R | R | R | R | R | S |
| MW-C48 | R | R | R | R | R | R | R | R | I | S | R | R | I | R | I | R | I | R | I | R | R | S | I | I | R | R | R | R | R | R |
| MW-C52 | R | R | R | R | R | R | R | R | I | I | R | R | R | R | R | R | R | R | R | R | R | S | S | I | R | R | R | R | R | R |
| MW-C58 | R | R | R | R | R | R | R | R | R | I | R | R | I | R | R | R | R | R | R | R | R | S | I | I | I | I | I | R | R | R |
| MW-C61 | R | R | R | R | R | R | R | R | I | I | R | R | R | R | R | R | R | R | R | R | R | S | R | I | R | R | R | R | R | R |
| MW-C63 | R | R | R | R | R | R | R | R | I | I | R | R | R | R | R | R | I | R | I | R | R | S | I | I | I | I | I | R | R | R |
| MW-C77 | R | R | R | R | R | R | R | R | R | I | R | I | R | R | R | R | R | R | I | I | I | S | I | I | R | I | R | R | R | R |
| MW-C79 | R | R | R | R | R | R | R | R | I | I | R | R | R | R | R | R | R | R | R | R | I | S | I | I | R | R | I | R | R | I |
| MW-C81 | R | R | R | R | R | R | R | R | I | I | R | R | R | R | R | R | R | R | R | R | I | S | I | I | R | R | R | R | R | R |
| MW-M10 | R | R | R | R | R | R | R | R | I | I | R | R | R | R | I | R | I | R | R | I | R | S | I | S | I | I | I | R | R | R |
| MW-M13 | R | S | S | R | R | I | S | I | S | I | R | I | R | R | I | R | I | R | I | I | S | S | S | R | R | S | I | R | R | R |
| MW-M14 | R | I | S | R | R | R | I | R | I | I | R | I | S | I | I | I | I | R | R | R | R | S | I | R | I | I | I | R | R | R |
| MW-M15 | S | S | S | S | S | S | S | S | S | I | R | I | R | R | I | R | I | R | S | S | S | S | S | R | I | S | I | I | I | I |
| MW-M17 | I | S | S | R | S | S | S | S | R | R | R | R | S | I | I | R | R | R | S | S | R | I | S | S | R | S | S | R | R | S |
| MW-M19 | S | S | S | S | S | S | S | S | S | I | R | I | I | R | I | S | I | I | S | S | S | S | S | S | R | S | S | S | S | I |
| MW-M20 | R | I | S | R | R | R | I | R | I | R | R | I | R | R | I | R | R | R | R | R | I | I | S | R | R | R | R | R | R | R |
| MW-M4 | R | R | R | R | R | R | I | R | I | R | R | I | I | I | I | R | R | R | R | R | R | R | R | I | R | R | R | R | I | R |
| MW-M5 | R | I | S | R | R | R | I | R | I | I | S | I | R | R | R | I | I | R | R | R | R | S | I | S | S | S | R | R | R | R |
| MW-M9 | R | S | S | S | R | I | S | I | S | I | R | I | S | I | S | R | I | R | R | S | S | S | S | R | I | R | I | R | R | R |
| SBW-C23 | R | R | R | R | R | R | R | R | I | I | R | R | R | R | R | R | I | R | R | R | I | S | R | R | R | R | R | R | R | R |

Note: S - sensitive to tested antibiotic; I - intermediate resistant to tested antibiotic; R - resistant to tested antibiotic.

**SB decomposition of cellulolytic marine bacterial strains** ***in vitro***

**Methods:** The cellulolytic marine bacterial strains were incubated in SB broth (g/L) containing 10 NaCl, 4 SB powder, 0.24 MgSO_4_, 0.011 CaCl_2_, 6.78 Na_2_HPO_4_, 3 KH_2_PO_4_, and 1 NH_4_C at 30°C for 15 days. The culture was filtered by sterilized neutral detergent (g/L) containing 37.2 EDTA, 13.6 sodium perborate, 30 SDS, 10 glycol ether (mL/L), and 23 Na_2_HPO_4_, and then washed by ethanol. The remaining SB (dry weight) in the culture was used to measure the rate of SB decomposition.

**Table S5**. The tests of SB decomposition of 28 cellulolytic marine bacteria *in vitro*.

|  | SB decomposition test | |
| --- | --- | --- |
|  | SBase | Decomposition rate (%) |
| CFW-C18 | 1.04 ± 0.27 | 63.81 ± 2.69 |
| CFW-C32 | 0.63 ± 0.24 | 27.62 ± 5.39 |
| CFW-C6 | 1.23 ± 0.54 | 13.81 ± 0.83 |
| CFW-C7 | 0.12 ± 0.14 | 36.47 ± 1.15 |
| CFW-C9 | – | – |
| MW-C42 | 0.70 ± 0.02 | 35.24 ± 3.73 |
| MW-C44 | 2.15 ± 0.16 | 2.59 ± 0.46 |
| MW-C45 | 0.02 ± 0.01 | 29.52 ± 3.34 |
| MW-C47 | – | – |
| MW-C48 | 1.44 ± 1.80 | 2.59 ± 0.46 |
| MW-C52 | 0.02 ± 0.23 | 35.23 ± 3.03 |
| MW-C58 | 0.55 ± 0.32 | 39.05 ± 0.60 |
| MW-C61 | 0.12 ± 0.18 | 0.00 |
| MW-C63 | 1.90 ± 0.90 | 33.33 ± 3.44 |
| MW-C77 | 3.75 ± 0.97 | 29.10 ± 6.81 |
| MW-C79 | 2.92 ± 0.43 | 0.00 |
| MW-C81 | 0.02 ± 0.12 | 35.44 ± 3.30 |
| MW-M10 | 0.22 ± 0.19 | 39.05 ± 1.09 |
| MW-M13 | 0.61 ± 0.75 | 36.29 ± 1.73 |
| MW-M14 | 0.44 ± 0.14 | 10.67 ± 1.15 |
| MW-M15 | 1.08 ± 0.58 | 48.57 ± 6.55 |
| MW-M17 | 1.75 ± 0.97 | 31.43 ± 4.90 |
| MW-M19 | 4.14 ± 0.89 | 29.52 ± 1.71 |
| MW-M20 | 1.03 ± 0.18 | 33.73 ± 3.54 |
| MW-M4 | 0.07 ± 0.10 | 2.72 ± 0.25 |
| MW-M5 | 0.62 ± 0.05 | 0.00 |
| MW-M9 | 0.50 ± 0.44 | 35.49 ± 1.56 |
| SBC-C23 | 0.16 ± 0.25 | 35.24 ± 6.44 |
| Control | – | 0.95 ± 1.65 |
